# Improvement of cognitive deficit of curcumin on scopolamine-induced Alzheimer’s disease models

**DOI:** 10.22088/cjim.13.1.16

**Published:** 2022

**Authors:** Güzin Çakmak, Davut Sinan Kaplan, Caner Yıldırım, Hasan Ulusal, Mehmet Tarakçıoğlu, Zeynel Abidin Öztürk

**Affiliations:** 1 Gaziantep University, Faculty of Medicine, Department of Internal Medicine, Division of Geriatric Medicine, Gaziantep, Turkey; 2 Gaziantep University, Faculty of Medicine, Department of Physiology, Gaziantep, Turkey; 3 Gaziantep University, Faculty of Medicine, Department of Biochemistry, Gaziantep, Turkey

**Keywords:** Cognitive dysfunction, Curcumin, Inflammation, Morris Water Maze Test, Scopolamine

## Abstract

**Background:**

It has been suggested that curcumin may be useful in diseases with cognitive dysfunction because it slows the progression and leads to the improvement of cognitive functions. In this study, the protective effects of curcumin on scopolamine-induced rat models of cognitive impairment were evaluated.

**Methods:**

21 male Wistar Albino rats, 1 year old, 200±25 grams, were included in the study. They were divided into three groups (n: 7 in each group); the untreated control group, scopolamine group, and the group treated with curcumin and then exposed to scopolamine. Animals were evaluated for behavioral tasks with the Morris Water Maze test. Interleukin-6 (IL-6), tumor necrosis factor-alpha (TNF-alpha), total oxidative status (TOS), and total antioxidative status (TAS) were measured in hippocampal tissues. CRP levels were measured in serum specimens.

**Results:**

We found that the length to reach the platform was the highest in the scopolamine group, and the lowest in the curcumin group (p<0.001). Time to reach the platform was the longest in the scopolamine group, and the shortest in the curcumin group (P=0.002). The length to reach the platform was the highest in the scopolamine group, and the lowest in the control group in the probe test (p<0.001). IL-6 levels were higher in the scopolamine group than the curcumin group (P=0.017) and the control group (P=0.005).

**Conclusion:**

We revealed that curcumin provides a protective effect on scopolamine-induced cognitive impairment mimicking Alzheimer's disease. The use of curcumin for the protection of cognition in individuals at risk of developing AD may be considered.

Memory and learning form the basis of cognitive functions. Cognitive dysfunction is frequently seen in neurodegenerative diseases, whose frequency increases with age. Dementia is one of the neurodegenerative diseases that cause impaired higher cortical functions (1). Irreversible and progressive cognitive dysfunctions characterize AD. Short-term memory loss, cognitive deficits, and decrement in daily living functions are common symptoms of AD (2). At the advanced stage of the disease, affected patients often withdraw from social life, lose body functions, and die. Studies have shown that AD shortens life expectancy (3). Approximately 46 million people are affected by dementia worldwide, and the number is projected to increase to 131.5 million by 2050. Because of the health and economic burden that it caused; it is essential to explore further therapies for AD. Neuronal loss, senile plaques, neurofibrillary tangles, and accumulation of cytosolic lipids are the main pathological features of AD (4). Amyloid-β (Aβ) peptide and tau proteins are also involved in AD pathophysiology by forming plaques and tangles.

The loss of synapses, and atrophy of the cerebral cortex are also seen in AD (5). Loss of acetylcholine secreting neurons in the nucleus Basilis of Meynert (nBM), an area that projects to the frontal cortex, is typical for AD (6). The brain, as a lipid rich organ and has a high oxygen demand, is susceptible to oxidative stress, and has peroxidation-susceptible cells (7). Impairments in mitochondrial function and oxidative stress are very important in aging and neurodegenerative diseases, especially AD .Curcumin is a polyphenol derived from turmeric (Curcuma longa), an herb, that grows in southern Asia (8). It has been used as a spice, a food coloring agent, and herbal medicine in traditional medicine practice (9). Amyloid-beta inhibition, cholesterol-lowering, copper-binding, tau inhibition, modulation of microglia, acetylcholine esterase inhibition, modification of insulin signaling pathway, and anti-oxidation are possible mechanisms that explain the effect of curcumin on cognitive functions (10). Scopolamine is a nonselective, and competitive inhibitor of the muscarinic acetylcholine receptor that is involved in working memory. One of the most used pharmacological models in learning and memory experiments is the scopolamine-induced memory disorder model. It blocks muscarinic acetylcholine receptors, and causes temporary blockage in neuronal signal transmission, and subsequently resulting in learning and memory disturbance (1). Scopolamine can cause cellular changes such as oxidative stress, mitochondrial dysfunction, apoptosis, and neuroinflammation (11). These pathological changes are similar to the changes seen in AD models. It is also used for developing AD models. The development of the scopolamine AD model is based on the cholinergic hypothesis of aging and AD (12). In this research, we evaluated the protective effects of curcumin on scopolamine-induced cognitive impairment models of rats. 

## Methods

**Animals:** 21 male Wistar Albino rats of 200 ± 25grams and 1-year-old were introduced to the study. Animals had unrestricted access to water and food sources. They were kept in a room with adjustable temperature (22± 2°C) on a 12 h/12 h light/dark cycle (lights on at 7:00 AM) and were randomly housed in groups of 7 in polypropylene cages with sawdust as bedding. The animal handling and experiments were performed following the institutional guidelines (Reference No: 151) by Gaziantep University Research Ethics Committee.

**Administration of curcumin and other chemicals:** The rats were divided into three groups (n:7 in each group); the untreated control group, scopolamine group, and the group treated with curcumin and then exposed to scopolamine. 200 mg/kg/day intraperitoneal (i.p) curcumin (Sigma Aldrich, USA) was given to the rats in the curcumin group for seven days (between days -7 and 0). Dimethyl sulfoxide (DMSO), which is a solvent of curcumin, was given to other groups at the same time (13). It has been shown in previous studies that curcumin applied intraperitoneally reaches its optimum level of efficacy in 7 days (14). Scopolamine hydrobromide (Sigma Aldrich, USA) was given to scopolamine and curcumin groups at a single dose, sixteen mg per kg i.p. on day 0 to create cognitive impairment models (15). Isotonic water, which is solvent for scopolamine, was given to the control group on day 0. In previous studies, memory impairment was observed at the 24th hour of scopolamine administration, and this effect was observed to increase at the 48th hour (16).

**Behavioral tests:**

**Spatial memory assessment: **Rats tested for spatial reference memory by the Morris water maze is a hidden platform task. We can base the logic of Morris Water Test on a scheduled hippocampus-dependent protocol (17). Morris water maze is a circle shaped pool, 120x50 cm in dimension. The pool is ﬁlled with water up to 25 cm and stained with methylene blue, and a 10x10 cm platform is placed 2 cm below the water. The platform was in the southeast quadrant (S3 zone). The water temperature was set at 24±2°C degrees. Four distal cues were positioned around the maze. Rats were exposed to a single training period each day, consisting of four sequential trials, with an inter-trial interval of 45 s (30 s spent on the platform and 15 s drying time). All trials (during acquisition and reversal) were 120 s in duration. In all trials, rats were released at the edge of the pool, in front of the cue, facing towards the wall. The same sequence of cues was used for all animals. For each trial, the time that the rat reached the platform, and the distance traveled for the platform was recorded. Rats that were not able to locate the platform within this time were guided towards the platform by the experimenter (18).

**Probe trial:** To evaluate memory reinforcing process, a probe test was done after 24 h of a 5-day test. In this test, the platform is removed from the maze. Rats were expected to swim more in S3 zone. The test aimed to measure the consolidation of memory. Four trials are made for each rat. Rats were dropped into the pool from 4 different points located between cues. The time that the rat reached the S3 zone and the previous location of the platform and the distance it traveled to reach those destinations were recorded (17).

**Isolation of brain tissues:** After the probe test, 45 mg per kg ketamine and 21 mg per kg xylazine were applied to rats via intramuscular injection for anesthesia. Then, they were euthanized with the guillotine (19). The hippocampus of rats was dissected, frozen. Blood specimens of rats were also taken, and their serums were separated. Specimens were kept in -80 °C chambers. 

**Biochemical tests:** Biochemical analysis was done after samples were dissolved in +4 °C one week after dissection and freezing of tissues of rats. Hippocampal tissue was homogenized with sonic homogenization. To determine inflammatory status and oxidative status in brain tissue, IL-6, TNF-alpha, TOS, TAS were measured. CRP levels were studied in serum samples. Measurement of CRP, IL-6, and TNF-alpha levels was done via ELISA method by using commercial kits (Rel Assay, Gaziantep, Turkey) on a Roche Hitachi Cobas c501 automatic analysis (Indianapolis, IN, USA). 

Measurements of TOS and TAS were done via the Erel method. We can base the logic of this test on the oxidation of iron in an acidic medium and the measurement of the ferric ion by xylenol orange. The assay is calibrated with hydrogen peroxide and the results are expressed in terms of micromolar hydrogen peroxide equivalent per liter (μmol H2O2 Equiv/L). The TOS/TAS ratio gives the oxidative stress index value (20).

**Statistics: **Normality analysis was done with Kolmogorov-Smirnov test. Data were shown as mean± standard error (SE) or standard deviation (SD). Kruskal-Wallis analysis was done to assess the overall statistical significance of differences among the tested groups (α=0.05). Comparison of behavioral test parameters and biochemical parameters were done by one-way ANOVA. Two-way ANOVA was used to evaluate the change in parameters according to days. Tamhane's T2 multiple comparison tests are used for the analysis of the difference between groups. Data analysis was done by SPSS Version 26.0.

## Results

Rats of the control group which were not treated, scopolamine group and treated with curcumin and then exposed to scopolamine group were trained on the standard, fixed location, covered escape platform, spatial reference memory version of the Morris water maze task. All rats swam well in the pool, and there was truly little evidence of floating behavior. In this study, we found that path length for the platform and path length for the zone where the platform was located (S3 zone) values were highest in the scopolamine group and lowest in the curcumin group (p<0.01). Latency to reach platform was the longest in the scopolamine group, and shortest in the curcumin group (P=0.002). 

The results are given in table 1. The comparison of scopolamine and curcumin groups with the control group is summarized in table 2. Path length for the platform, path length for S3 zone and latency to reach the platform was longer in the control group than the curcumin group. Path length for the platform and S3 zone of the control group is shorter than the scopolamine group. 

In the probe test, path length for the platform was highest in the scopolamine group, lowest in the control group (p<0.001). The results were summarized in table 3. The comparison of probe test results of rats in scopolamine and curcumin groups with the control group is summarized in table 4. 

When we evaluated the change of behavior test parameters with time, we noticed path length to platform and S3 zone started to diminish on day 3 or 4 in the rats. There was a significant decrement in path length to reach the platform in the curcumin group on day 3 (P=0.016) and the control group on day 4 (P=0.007). There was no significant decrement in the scopolamine group. There was also a significant decrement in path length for S3 zone in the curcumin group on day 3 (P=0.025) and the control group on day 4 (P=0.013). 

**Figure 1:**
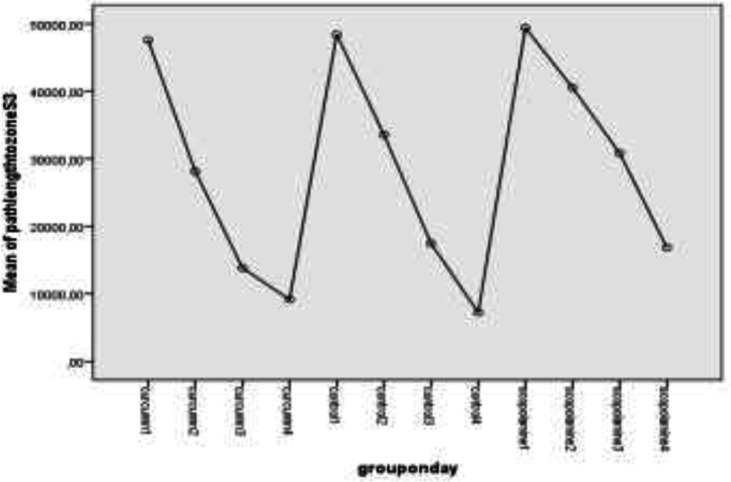
Path length to the platform according to days

**Figure 2:**
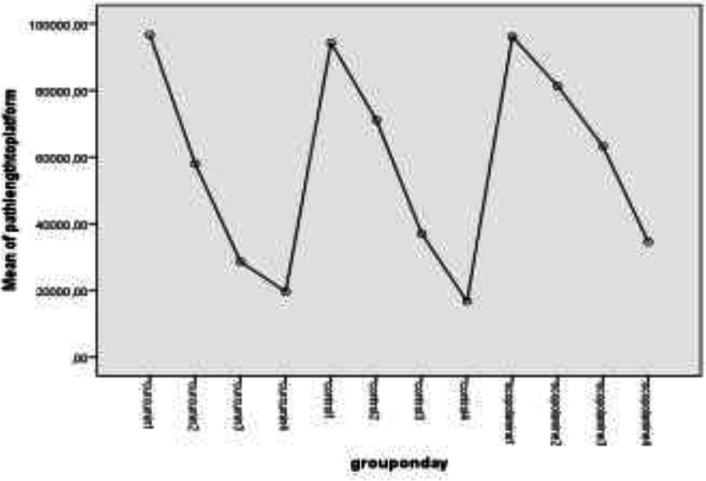
Path length to S3 zone according to days

There is no decrease in the scopolamine group in this parameter too. The values of parameters on different days were summarized in table 5. Figures 1 and 2 show the changes in path lengths to reach the platform and S3 zone on days 1 to 4, respectively. 

IL-6 levels were highest in the scopolamine group, lowest in the control group (P=0.007). IL-6 levels were higher in scopolamine group than both the curcumin group (P=0.049) and the control group (P=0.018). TNF-α levels were also significant. 

The oxidative stress index was slightly high in the scopolamine group than the others, but there was no statistical significance either. CRP levels of rats were extremely low (minimum: 0.1 mg/dl; maximum:0.4 mg/dl), and any significant difference between groups was not observed (P=0.258). The results were summarized in table 6. 

**Table 1:** Comparison of behavior test parameters according to groups (n=7)

**Parameter**	**Curcumin** **Mean±SD**	**Control** **Mean±SD**	**Scopolamine** **Mean±SD**	**p**
Path length to the platform (cm)	30.06**±**1.4	34.53**±**1.2	37.9**±**0.45	<0.001*
Path length to S3 zone (cm)	14.5**±**0.54	16.8**±**0.64	18.6**±**0.31	**<**0.001*
Latency to the first platform (s)	18.94**±**1.92	27.25**±**2.4	29.7**±**2.42	0.002*
Latency to S3 zone(s)	3.06±0.55	4.1±0.55	2.7±0.5	0.152

cm: centimeter

s: second

SE: standard error

**Table 2:** Comparison of groups (n=7) with the control group (n=7)

**Parameter**	**Curcumin ** **Control**	**Scopolamine ** **Control**
Path length to the platform (cm)	p=0.049*	p=0.035*
Path length to S3 zone (cm)	p=0.02*	p=0.047*
Latency to the first platform (s)	p=0.022*	p=0.858
Latency to S3 zone(s)0	p=0.431	p=0.171

cm: centimeter

s: second

**Table 3:** Comparison of probe test parameters according to groups (n=7)

**parameter**	**Curcumin ** **Mean±SD**	**Control ** **Mean±SD**	**Scopolamine** **Mean±SD**	**p**
Path length to the platform (cm)	48.2**±**0.21	44.15**±**0.98	55.70**±**1.18	<0.001*
Path length to S3 zone (cm)	23.25**±**2.69	19.9**±**2.5	27.9**±**1.12	0.061
Latency to the first platform (s)	11.82**±**4.65	18.31**±**3.6	30.40**±**7.13	0.071
Latency to S3 zone(s)	5.14**±**1.8	9.15**±**2.05	7.12**±**1	0.783

cm: centimeter

s: second

SD: standard deviation

**Table 4:** Comparison of other groups (n=7) with the control group (n=7)

**Parameter**	**Curcumin Control**	**Scopolamine ** **Control**
Path length to the platform (cm)	p=0.017*	p<0.001*
Path length to S3 zone (cm)	p=0.761	p=0.055
Latency to the first platform (s)	p=0.651	p=0.482
Latency to S3 zone(s)	p=0.424	p=0.783

cm: centimeter

s: second

**Table 5:** Parameters according to days

**Parameter**	**Group**	**Day**	**mean** **±** **SD**
Path length to the platform (cm	Curcumin	1	96.6**±**69.22
		2	57.9**±**67.05
		3	28.7**±**44.88
		4	19.7**±**32.22
	Control	1	94.22**±**68.64
		2	71.12**±**57.66
		3	37.07**±**37.35
		4	16.8**±**16.31
	Scopolamine	1	96.1**±**64.01
		2	81.3**±**61.49
		3	63.2**±**64.69
		4	34.6**±**54.34
Path length to S3 zone (cm)	Curcumin	1	47.6**±**36.08
		2	28.14**±**35.02
		3	13.84**±**22.44
		4	9.16**±**16.11
	Control	1	48.4**±**38.87
		2	33.58**±**28.75
		3	17.46**±**17.40
		4	7.23**±**7.74
	Scopolamine	1	49.37**±**34.98
		2	40.53**±**33.16
		3	30.81**±**31.99
		4	16.87**±**29.52

cm: centimeter

SD: standard deviation

**Table 6:** Relationship between groups and inflammatory/oxidative stress parameters

	**Curcumin (n=7)** **Mean±SD**	**Control (n=7)** **Mean±SD**	**Scopolamine (n=7)** **Mean±SD**	**p**
IL6(mg/dl)	11.29±0.58	10.17±0.8	13.41±0.5	0.007*
TNF-α (mg/dl)	22.57±2.01	22.2±1.1	23.3±1.1	0.869
OSI	18.9±1.6	20.7±1.5	22.1±2.08	0.436
CRP	0.2±0.08	0.16±0.08	0.13±0.08	0.258

IL-6: Interleukin-6, TNF-α: Tumor necrosing factor-alpha

mg/dl: milligram/deciliter, CRP: C-reactive protein SE: standard error

## Discussion

We aimed to evaluate the protective effect of curcumin treatment in spatial memory, consolidation memory, inflammatory status, and oxidative stress in the cognitive impairment models generated with intraperitoneal scopolamine injection. According to the results of the current study, i.p. scopolamine seemed to induce significant spatial memory deterioration, as evidenced by increased distances to move to reach the platform and S3 zone and increased latency to reach the platform. A decrement in consolidation memory was also seen in the probe test. We can say that these changes in the scopolamine group mimic Alzheimer's disease. It has also been elicited in the present study that intraperitoneal administration of scopolamine causes an increase in inflammation, which might be responsible for cognitive impairment. Ahmad et al. revealed that scopolamine administration for 14 days increased the levels of IL-1β and TNFα, and an increase in pro-inflammatory cytokines affected memory impairment (21). 

Unlike this study, we showed the proinflammatory effect of single-dose scopolamine on day 6. Like this study, we revealed an increment in the level of hippocampal IL-6, a proinflammatory cytokine, in the scopolamine group. We also observed a statistically insignificant increase in TNF-α levels in the hippocampal tissues. Another study also proposed that competitive inhibition of muscarinic receptors by scopolamine might be the cause of an increment in the expression of TNF-α in the hippocampus (22). Fan et al. revealed that scopolamine inactivates superoxide dismutase (SOD) and glutathione peroxidase (GSH-Px), and inhibits ATPase enzyme activity in the hippocampus and cerebral cortex. All of these causes oxidative damage (23). We also observed a statistically insignificant increase in the OSI value of the hippocampus tissue of the scopolamine group, which is the measure of oxidative stress. 

In our study, we revealed that i.p. curcumin 200 mg/kg/day for seven days protected the spatial and consolidation memory from the effect of scopolamine. The length to move to reach the platform and S3 zone and latency to reach the platform was the shortest in the curcumin group when compared to both the control group and the scopolamine group. We saw a significant decrement in both lengths to reach the platform and S3 zone within days in the curcumin and control group. The improvement was seen earlier in the curcumin group than the control group. There was no improvement in the scopolamine group within days. We also showed the protective effect of curcumin on consolidation memory with a probe test. Agrawal et al. evaluated the preventive and therapeutic effect of 200 mg/kg curcumin on sporadic dementia caused by scopolamine. They administered curcumin orally 14 days before disease induction or six days after its induction. Similar to our study, they used the Water Morris Maze Test for behavioral assessment. It has been observed that curcumin has not only therapeutic properties on cognitive dysfunction but also preventive function as in our study (24). Awasthi et al. studied the preventive and therapeutic role of curcumin at oral doses of 10, 20, and 50 mg/kg. They revealed that curcumin prevented memory loss at dosages of 20 and 50 mg/kg in a dose-dependent manner (25). 

Zhang et al. evaluated the protective effects of curcumin at 50, 100, and 200 mg/kg, i.p, on intraventricularly injected dementia models. They set out that protective treatment (7-day administration) with 200 mg/kg, i.p. curcumin prevented cognitive deficits compared to placebo (26). The result of this study is compatible with our study. Wu et al. demonstrated that curcumin is protective against surgery-induced cognitive dysfunction in aged mice. It was observed that curcumin provided this function by preventing the antioxidant enzyme activity induced by surgery and increasing the synthesis of brain-derived neurotrophic factor (BDNF) and pAkt. Curcumin also neutralized the activity of choline acetyltransferase induced by surgery. This study shows that curcumin prevents cognitive dysfunction through many pathways (27). 

Xu et al. showed that curcumin prevents brain damage and cognitive dysfunction related to brain ischemia-reperfusion injury. Curcumin has also been shown to be effective in reducing the tumor necrosis factor (TNF)-α, interleukin (IL)-6, IL-1β, reactive oxygen species (ROS), and malondialdehyde (MDA) levels and increasing the activity of superoxide dismutase (SOD) and catalase (CAT) in that study (28). Reeta et al. revealed that cognitive impairment associated with phenobarbitone and carbamazepine could be ameliorated by curcumin. Unlike our study, in this study, agents that cause cognitive dysfunction and curcumin were given simultaneously, and it was investigated whether it could heal cognitive dysfunction (29). The results of all these studies were compatible with our work. According to the results of all these studies, we can conclude that curcumin can be effective in both preventing and improving cognitive dysfunction.

Our study is a unique study that showed the protective effect of i.p. administered curcumin on memory impairment in cognitive disturbance rat models that formed by single-dose i.p. scopolamine injection. It is an undeniable fact that this methodology is less disturbing for experimental animals. We showed the effect of curcumin on both spatial and consolidation memory. It could be better to evaluate the anxiety levels of rats with the elevated plus-maze test We obtained a significant result regarding IL-6 levels. However, the differences in other inflammation and oxidative stress parameters were not statistically significant. Working with a low number of subjects may be the reason for this. It could be a choice to evaluate memory by the object recognition test, which is less stressful for rats, and this would result in less impact on oxidative stress and inflammation parameters, too (30). It could also be better to add groups into the study, which were given standard treatments as donepezil or memantine. 

In this study, we concluded that curcumin could prevent cognitive damage due to scopolamine. Treatment of cognitive disturbance in several neurodegenerative diseases is open to innovations due to a lack of definitive treatment. Studies on this topic suggest that the role of nutritional supplements like curcumin may be important in both prevention and treatment. More investigation is necessary on this subject.

## Author Contributions:

Conceptualization: GC, DSK, CY, ZAO Resources: GC Writing—original draft preparation: GC Methodology: DSK, CY, GC, HU, MT Writing—review and editing: GC and ZAO Supervision: DSK, MT, ZAO

## Funding:

This research received no specific grant from any funding institution or agency in the public, commercial, or non-profit sectors.

## Conflict of Interest Statement:

The authors declare no conflicts of interest.
